# Comparison of heart, grace and TIMI scores to predict major adverse cardiac events from chest pain in a Spanish health care region

**DOI:** 10.1038/s41598-023-44214-3

**Published:** 2023-10-12

**Authors:** Iris N. San Román Arispe, Joaquim Sol, Ana Celma Gil, Javier Trujillano, Marta Ortega Bravo, Oriol Yuguero Torres

**Affiliations:** 1 Centre d’ Urgències en AtencióPrimària. InstitutCatalà de La Salut (ICS), Lleida, Spain; 2 Multidisciplinary Research Group in Primary Care Therapeutics and Interventions (RETICAP), Fundació Institut Universitari per a La Recerca a l’Atenció Primària de Salut Jordi Gol I Gurina (IDIAPJGol), Lleida, Spain; 3 Atenció Primària, Institut Català de La Salut, Lleida, Spain; 4 Metabolic Physiopathology Research Group, Experimental Medicine Department, Lleida University-Lleida Biochemical Research Institute (UdL-IRB Lleida), Lleida, Spain; 5 Research Support Unit, Fundació Institut Universitari recerca l’Atenció Primària Salut Jordi Gol i Gorina (IDIAPJGol), Lleida, Spain; 6 Intensive Care Department, Hospital Universitari Arnau de Vilanova, Lleida, Spain; 7 Medicine and Surgery Department, Universidad de Lleida, Lleida, Spain; 8 Centro de Atención Primaria Almacelles, InstitutCatalà de La Salut (ICS), Lleida, Spain; 9 Clinical Ultrasound Research Group in Primary Care (GRECOCAP), Fundació Institut Universitari Per a La Recerca a l’Atenció Primària de Salut Jordi Gol i Gurina (IDIAP Jordi Gol), Lleida, Spain; 10 ERLab Research On Emergencies, IRB Lleida, Lleida, Spain

**Keywords:** Cardiology, Health care, Medical research

## Abstract

Acute non-traumatic chest pain (ANTCP) is the second cause of consultation in the Emergency department (ED). About 70% of all Acute Myocardial Infarctions present as non persistent ST-elevation acute coronary syndrome (NSTE-ACS) in the electrocardiogram. Our aim was to compare whether the HEART risk score is more effective than the GRACE and TIMI scores for the diagnosis and prognosis of Major Adverse Cardiac Events (MACE) at six weeks in patients with ANTCP and NSTE-ACS. A prospective cohort study was conducted with patients with ANTCP that attended an ED and a Primary Care Emergency Center (PCEC) from April 2018 to December 2020. The primary outcome was MACE at six weeks. Diagnostic performance was calculated for each scale as the Area under the Receiver Operating Characteristic (ROC) curve (AUC), sensitivity (SE), specificity (SP), and predictive values (PV). Qualitative variables were compared using the Chi-square test, and continuous variables were compared using the nonparametric Kruskal–Wallis test. We adjusted a logistic regression for risk groups, age, and gender to determine the effect of the HEART, GRACE, and TIMI scores on MACE. The degree of agreement (kappa index) was calculated in the categorical classification of patients according to the three risk scales. Cox proportional hazards regressions were performed for each scale and were compared using partial likelihood ratio tests for non-nested models. From a sample of 317 patients with ANTCP, 14.82% had MACE at six weeks. The AUC was 0.743 (95% CI 0.67–0.81) for the HEART score, 0.717 (95% CI 0.64–0.79) for the TIMI score, and 0.649 (95% CI 0.561–0.738) for the GRACE score. The HEART scale identified low-risk patients with a higher SE and negative PV than the GRACE and TIMI scores. The HEART scale was better than the GRACE and TIMI scores at diagnosing and predicting MACE at six weeks in patients with ANTCP and probable NSTE-ACS. It was also a reliable tool for risk stratification in low-risk patients. Its application is feasible in EDs and PCECs, avoiding the need for complementary tests and their associated costs without compromising patient health.

## Introduction

Acute non-traumatic chest pain (ANTCP) is the second cause of consultation in emergency departments (ED) in industrialized countries. About 70% of patients with ANTCP who develop major adverse cardiac events (MACE) present as a non persistent ST-elevation acute coronary syndrome (NSTE-ACS) in the electrocardiogram (ECG). Managing ANTCP suspected of NSTE-ACS correctly in EDs and primary care emergency centers (PCECs) is essential to avoid inappropriate discharges and costs related to complementary tests and unnecessary admissions^1–3^.

On the one hand, inadequate discharges lead to new consultations for ANTCP, and some patients end up developing MACE within six weeks. In a study conducted in the US in 2010^4^, 5–10% of patients were discharged from the ED because of non-coronary ANTCP, and in subsequent visits they were diagnosed with an acute myocardial infarction (AMI).

On the other hand, Owens et al.^4^observed that 20% of the consultations for ANTCP and probable NSTE-ACS result in acute coronary syndrome (ACS). Half of the patients admitted for suspected ACS who underwent diagnostic cardiac tests were ultimately not diagnosed with ACS. These tests amount to 10 billion dollars/year,or 3,000–6,000 dollars/patient,butonly 10% of these indicate a cardiac issue.

In Spain^2^, ACS is one of the main causes of morbidity, mortality, and costs^2^. There are approximately 120,000 cases of ACS per year, 41.67% of which are hospitalized with NSTE-ACS.

Finally, because diagnosing MACE in the context of an NSTE-ACS is complex, it is important to have a rapid and reproducible tool for both high-intermediate-risk patients, who require additional tests, and low-risk patients, who can be discharged quickly and safely.

In an attempt to improve theaccuracy in the diagnosis of MACE in patients with probable NSTE-ACS, the European Society of Cardiology (ESC)^3^ and the National Institute for Health and Care Excellence (NICE)^5^ recommend the use of the Global Registry of Acute Coronary Events (GRACE 2.0)^6^ and the thrombolysis in myocardial infarction (TIMI) risk scores^8^. However, according to validation studies, these recommendations are more useful in patients with a high probability of presenting ACS^10,11,14^.

To help identify low-risk patients, Six AJ et al. (2008)^7^ developed the medical history, ECG, age, risk factors, and troponin (HEART) score,a risk stratification tool for MACE in the context of NSTE-ACS. In 2013 Backus et al.^9^ externally validated the HEART score in a prospective multicenter study, in which the c-statistic of the HEART score (0.83) was significantly higher than that of the TIMI (0.75) and GRACE (0.70) respectively (; *p* < 0.0001) scores.In their systematic review and meta-analysis, Ke J et al. (2021)^26^ observed that TIMI and HEART were superior to GRACE for predicting MACE risk in acute chest pain patients admitted to the ED.

Our aim was to prospectively compare the performance of the HEART, TIMI, and GRACE scores relative to the diagnosis and prognosis of MACE at six weeks in patients presenting with ANTCP and suspected NSTE-ACS in the ED of a Hospital and of a PCEC in Lleida, Spain.

## Methods

### Design

To compare the HEART, TIMI, and GRACE scores, we performed a prospective cohort study in patients treated in the ED of the Hospital Universitari Arnau de Vilanova (HUAV) and in the PCEC (CUAP) of Lleida, in the period from April 2018 to December 2020. The HUAV is the reference public hospital center of the province of Lleida and some regions of the strip of Aragón, and the CUAP is the PCEC of the city of Lleida, Spain. Both span a healthcare region of 350,000 inhabitants. The ED of the HUAV is a second-level hospital with a fully equipped laboratory that can measure cardiac troponin I (TnI) levels, liver profile, kidney function, coagulation, creatine kinase (CK), amylase serum, C-reactive protein (PCR), D-dimers (DD), B-type natriuretic peptide (pro BNP), and other blood tests. It also offers radiography service and radiological tests, including computed axial tomography (TAC) pulmonary angiography, Doppler ultrasound of lower extremities, abdominal ultrasound, echocardiogram, and cardiac magnetic resonance (RM). The cardiovascular service is available 365 days a year, 24 h a day, and performs percutaneous coronary intervention (PCI) and coronary artery bypass grafting (CABG).The CUAP is a PCEC in Lleida, Spain. It has a basic laboratory that can measure cardiac troponin T (cTnT) and DD and a radiography service. It is open 365 days a year, 24 h a day, and takes care of low- and medium-complexity health problems. High-complexity emergency pathologies are referred to the HUAV.

This was a double-blind study: the patients didn’t know to which risk group they were assigned or the treatment they received, and the doctors caring for the patients also didn’t know to which risk group the patients belonged. Neither the decisions nor the administered treatments were influenced by the risk stratification of the HEART, GRACE, or TIMI scores. The patients received their usual care without influence from the observational study.

The sample of 317 patients received the usual medical care in the ED and in the PCEC. In turn, without influencing the behavior of the physician attending the patient in the consultation, the patients were stratified according to the HEART, GRACE, and TIMI scores.

### Recruitment and selection of study subjects

Of all the patients who attended the HUAV ED and the PCEC with ANTCP, we selected those over 18 years of age who had ANTCP for more than five minutes; presented clinical characteristics compatible with suspected ACS according to the ESC guidelines^3^, with probable NSTE-ACS; signed the informed consent form; and had all the data to apply the three scales. We excluded patients who did not meet these criteria, and/or had a clear diagnosis of non-cardiac pathology; moved outside of Lleida during the six-week follow-up; modified any of the risk factors for acute cardiovascular events (e.g., initiation of cocaine use or any other cardioactive or vasoactive drug that directly produces and/or favors the development of MACE).

### Sample size calculation

An inclusion of 300 patients in the sample is the minimum to determine a difference in the C statistic for the Receiver Operating Characteristic curves (ROC curve) of 0.08, assuming a C statistic for the HEART scale of 0.83 with a type I error of 0.05.

**Figure 1:**
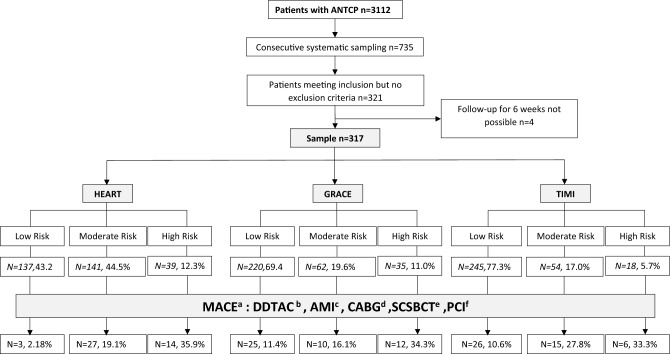
Flow chart of the patients included in the study.a: major acute cardiac event; b: death due to any cause; c: acute myocardial infarction; d:coronary artery bypass grafting; e: significant coronary stenosis but conservative treatment; f: percutaneous coronary intervention.

We obtained a representative sample of 317 patients from the study population through consecutive simple aleatory sampling, including those patients who met all the inclusion criteria and none of the exclusion criteria (Fig. 1). Patient follow-up was retrospective, based on the computerized and electronic medical records of the patients in the Catalan Health Institute’s database, ICS–Lleida (ECAP), routinely used by all primary care. Patient follow-up was also based on the clinical file system–Administrative Management of Patients (SAP) of the Catalan Health Institute’s (ICS) of the HUAV.

### Variables

Data on HEART risk score variables, including medical history, ECG, age, risk factors, and troponin, were collected (Table 1). The clinical history of chest pain with suspicion of ACS followed the ESC^3^ and NICE^5^ guidelines. The TnI test used in the HUAV was scored as follows: 0 points = normal, limit < 0.01 ng/mL; 1 point = 1–3 times the normal limit, 0.01–0.03 ng/mL; 2 points = more than 3 times the normal limit, ≥ 0.04 ng/mL. The cTnT test used in the PCEC was scored as follows: 0 points ≤ 40 ng/L; 1 point ≥ 40 and < 120 ng/L; and 2 points ≥ 120 ng/L. Both troponins are the upper limit of the reference range and correspond to the 99th percentile of the population studied by our method, without correction for hemolysis^3,10,17,24^. According to these items, patients were stratified into three risk levels: low (≤ 3 points), moderate^4–6^ points, and high (≥ 7 points).

**Table 1:** Variables of the risk scales and MACE at 6 weeks.

MACE^a^	No	Yes	Overall p
	N = 270	N = 47	
**Demographic variables**			
Age: variable present in HEART, GRACE and TIMI	57.6 (16.9)	63.6 (14.0)	0.012
65 years or over:			0.245
No	182 (67.4%)	27 (57.4%)	
Yes	88 (32.6%)	20 (42.6%)	
Sex:			0.107
Man	177 (65.6%)	37 (78.7%)	
Woman	93 (34.4%)	10 (21.3%)	
**Domicile:**			0.803
Rural	112 (41.5%)	21 (44.7%)	
Urban	158 (58.5%)	26 (55.3%)	
**Education:**			0.064
Higher Degree (Bachelor's Degree)	41 (15.2%)	13 (27.7%)	
Intermediate grade (technician, auxiliary, operator)	104 (38.5%)	12 (25.5%)	
Unskilled worker (laborer)	125 (46.3%)	22 (46.8%)	
**Employment status**			0.646
Employed	151 (55.9%)	24 (51.1%)	
Unemployed	119 (44.1%)	23 (48.9%)	
**Family status:**			0.318
Alone	27 (10.0%)	2 (4.26%)	
Couple	154 (57.0%)	26 (55.3%)	
Family	81 (30.0%)	19 (40.4%)	
Others, not relatives	8 (2.96%)	0 (0.00%)	
**Cardiovascular risk factors**			
History of chest pain: variable present in HEART			< 0.001
High Suspicion	71 (26.3%)	29 (61.7%)	
Medium Suspicion	132 (48.9%)	10 (21.3%)	
Low Suspicion	67 (24.8%)	8 (17.0%)	
**Smoking cigarettes: variable present in HEART and TIMI**			< 0.030
Yes	65 (24.1%)	19 (40.4%)	
No	205 (75.9%)	28 (59.6%)	
**Hypercholesterolemia: variable present in HEART and TIMI**			< 0.373
Yes	110 (40.7%)	23 (48.9%)	
No	160 (59.3%)	24 (51.1%)	
**Hypertension: variable present in HEART and TIMI**			< 0.016
Yes	135 (50.0%)	33 (70.2%)	
No	135 (50.0%)	14 (29.8%)	
**Diabetes: variable present in HEART and TIMI**			< 0.005
Yes	46 (17.0%)	17 (36.2%)	
No	224 (83.0%)	30 (63.8%)	
**Dyslipidemia: variable present in HEART and TIMI**			< 0.287
Yes	112 (41.5%)	24 (51.1%)	
No	158 (58.5%)	23 (48.9%)	
**Coronary sten. (> 50%)**^b^**: variable present in TIMI**			< 0.038
Yes	26 (9.63%)	10 (21.3%)	
No	244 (90.4%)	37 (78.7%)	
TAS: variable present in GRACE	142 (22.2)	143 (22.8)	< 0.820
**F. history of ischemic heart disease (M < 55 y. W < 65 y.) **^c^**: variable present in HEART and TIMI**			< 0.018
Yes	31 (11.5%)	12 (25.5%)	
No	239 (88.5%)	35 (74.5%)	
**Obesity: variable present in HEART**			< 0.628
Yes	68 (25.2%)	14 (29.8%)	
No	202 (74.8%)	33 (70.2%)	
**Risk factors: variable present in HEART and TIMI**			< 0.001
= 3	74 (27.4%)	27 (57.4%)	
1–2	139 (51.5%)	16 (34.0%)	
No risk factors	57 (21.1%)	4 (8.51%)	
**KILLIP**			
KILLIP^d^: variable present in GRACE			< 0.001
1	253 (93.7%)	34 (72.3%)	
2	15 (5.56%)	11 (23.4%)	
3	2 (0.74%)	1 (2.13%)	
4	0 (0.00%)	1 (2.13%)	
**ECG**			
ECG: variable present in HEART and GRACE			< 0.001
Significant ST depression	9 (3.33%)	5 (10.6%)	
Unsp. repolarization alteration^e^	88 (32.6%)	24 (51.1%)	
Normal	173 (64.1%)	18 (38.3%)	
**Troponin**			
Troponin: variable present in HEART and TIMI			< 0.001
= 3 times the normal limit	15 (5.56%)	7 (14.9%)	
1–3 times the normal limit	20 (7.41%)	15 (31.9%)	
= normal limit	235 (87.0%)	25 (53.2%)	

a: major adverse cardiovascular events; b: history of ischemic heart disease (M < 55 y. W < 65 y.): Family history of ischemic heart disease in men under 55 years of age and in women under 65 years of age; c: coronary stenosis greater than 50%. Risk factors: cardiovascular risk factors; d: Killip-Kimball classification; e: unspecific alteration of repolarization on the electrocardiogram.

The variables for the GRACE 2.0 (age, heart rate, blood pressure, creatinine, Killip class, cardiorespiratory arrest on admission, elevated cardiac enzymes, ST segment elevation) and TIMI (age > 65 years, cardiovascular risk factors, coronary stenosis > 50%, two episodes of Angina in < 24 h, taking acetylsalicylic acid in the last 7 days, elevated creatine kinase isoenzyme MB (CK-MB) or troponin) scales were collected (Table 1). For both scales, for the “elevation of markers of myocardial damage,” we used the values TnI > 0.01 ng/mL and cTnT > 40 ng/L, respectively^3,10,11,17,24^.

Other independent variables were also considered, including sex, diagnoses at discharge, diagnoses at hospital admission [according to International Statistical Classification of Diseases and Related Health Problems 10th Revision (ICD-10)]^18^, cardiac tests performed, blood count and coagulation used in the diagnostic process, type of domicile (rural or urban), level of education (higher degree/bachelor's degree, intermediate degree/technician/auxiliary/operator, and unskilled worker/laborer), employment status (if the patient works or not), family status (if the patient lives alone or with a partner, and/or with children, and/or with parents, and/or with siblings), number of hours of hospital stay under observation in the ED or PCEC, numberof days of admission/hospital stay for suspected ACS, number of new visits for ANTCP in the following six weeks.

The dependent variable was MACE, which includes death due to any cause (DDTAC); AMI, defined according to the third universal definition of myocardial infarction^17^; PCI; CABG; and significant coronary stenosis but conservative treatment (SCSBCT) because of other concurrent pathologies that contraindicate any interventional coronary revascularization^9,25,27^.

### Data sources

We obtained the data for this study from the computerized registry of the SAPof the HUAV and ECAP, ICS—Lleida. The collected data were entered into the RED Cap (Research Electronic Data Capture) web platform.

### Statistical analysis

The diagnostic accuracy and Rand balanced accuracy of the HEART, GRACE, and TIMI scores in predicting MACE were calculated at the initial visit and six weeks later. This endpoint was chosen because the HEART score predicts the risk of MACE at 6 weeks^9^.

Continuous variables are expressed as the mean and 95% confidence interval (CI) and categorical variables as the absolute and relative frequency. Qualitative variables were compared using the Chi-square test, and continuous variables were compared using the nonparametric Kruskal–Wallis test. To determine the effect of the HEART, GRACE, and TIMI scores on MACE, we adjusted a logistic regression for risk groups, age, and gender. The degree of agreement (kappa index) was calculated in the categorical classification of patients according to the HEART, GRACE, and TIMI scores.The AUC was used as a discrimination model and precision index. The Hosmer-Lemeshowc-test was applied to assess model calibration by grouping patients by similar model results. The sensitivity (SE), specificity (SP), positive predictive value (PPV), negative predictive value (NPV), false positive rate (FPR), and false negative rate (FNR) were studied simultaneously. DeLong's test was used for testing two correlated ROC curves. Cox proportional hazards regressions were performed for each scale and were compared using partial likelihood ratio tests for non-nested models. A significance level of 0.05 (α = 0.05) was considered. All statistical tests were performed using R software version R-4.1.1.

During the study, patients that could not be followed up for six weeks, because of lack of data or impossibility of contact, were excluded from the analysis. To minimize this possible bias, a comparative analysis was made between the patients who were followed up and those who were not. When we detected clinically relevant differences, weights were assigned based on these differences. The weights were developed using the *Inverse probability weighting* algorithms^24^ that have been validated and applied in different observational studies.

### Ethics approval and consent to participate

According to Law 14/2007 concerning the Biomedical Research Regulations in Spain, Article 3-m, this study is an «Observational Study»: a study carried out on individuals in which the treatment or intervention to which they may be subjected is not modified nor is any other guideline prescribed that could affect their personal integrity. The patients received the optimal treatment according to the current guidelines of the HUAV and the CUAP of Lleida, Spain. All methods **were** performed in accordance with the relevant guidelines and regulations. The research was carried out in accordance with the Declaration of Helsinki, revised in 2013. The study was approved by the local ethics committee of the HUAV (minutes: 6/2017) and by the ComitèÈticd'InvestigacióClínica de l'IDIAP Jordi Gol (code: P17 / 219). All patients signed an informed consent form before participating.

## Results

Figure 1 shows the flow of patients in the study. A total of 3112 patients attended the HUAV ED and PCEC for ANTCP. Using consecutive systematic sampling and after applying the inclusion, exclusion, and follow-up loss criteria, we obtained a representative sample of 317 patients. The mean age was 58 years (Table 1), and 67.5% of them were male.

Table 1 shows the variables that were associated with MACE: 48.9% (*p* < 0.001) of patients had a clinical history of chest pain which was moderately suspicious; 50.0% (*p* = 0.016) were hypertensive; 93.7% (*p* < 0.001) had a Killip class 1; 64.1% (*p* < 0.001) had a normal ECG; 87.0% (*p* < 0.001) had troponin within normal limits; 51.5% (*p* < 0.001) had one or two cardiovascular risk factors; 78.7% (*p* = 0.107) were men; 57.4% (*p* < 0.012) were under 65 years of age;55.3% lived in urban areas (*p* < 0.803); 46.8% (*p* < 0.064) had an education level equivalent to an unskilled worker; and 55.3% (*p* < 0.318) lived with their partner.

**Figure 2:**
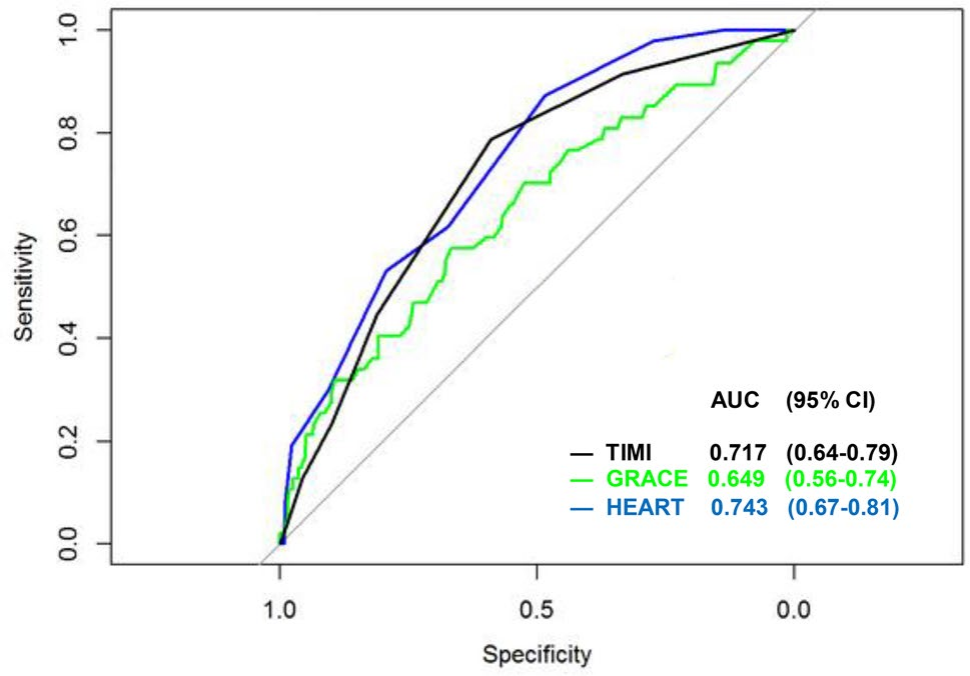
ROC curves for the HEART, TIMI, and GRACE risk scales.

The HEART score had the highest AUC (0.743; 95% CI: 0.674–0.812; Fig. 2).

Overall, 13.8% of patients had MACE at six weeks: 17.4% in the PCEC and 15.4% in the HUAV ED. MACE were most frequent in patients classified as high-risk according to the HEART score (35.9%; with a 95% CI: 21.20–52.82), followed by high-risk patients according to the GRACE (34.3%; with a 95% CI: 19.13–52.21) and TIMI scales (33.3%; with a 95% CI:13.34–59.01; Table 2).

**Table 2:** MACE according to the risk stratification of the HEART, GRACE, and TIMI scales.

ALL		HEART				GRACE			TIMI		
	[ALL]	N	High	Low	Moderate	High	Low	Moderate	High	Low	Moderate
	N = 317		*N* = *39*	*N* = *137*	*N* = *141*	*N* = *35*	*N* = *220*	*N* = *62*	*N* = *18*	*N* = *245*	*N* = *54*
DDTAC ^a^:		317									
Yes	7 (2.21%)		4 (10.3%)	2 (1.46%)	1 (0.71%)	6 (17.1%)	0 (0.00%)	1 (1.61%)	3 (16.7%)	2 (0.82%)	2 (3.70%)
No	310 (97.8%)		35 (89.7%)	135 (98.5%)	140 (99.3%)	29 (82.9%)	220 (100%)	61 (98.4%)	15 (83.3%)	243 (99.2%)	52 (96.3%)
AMI^b^:		317									
Yes	29 (9.15%)		8 (20.5%)	3 (2.19%)	18 (12.8%)	4 (11.4%)	17 (7.73%)	8 (12.9%)	2 (11.1%)	18 (7.35%)	9 (16.7%)
No	288 (90.9%)		31 (79.5%)	134 (97.8%)	123 (87.2%)	31 (88.6%)	203 (92.3%)	54 (87.1%)	16 (88.9%)	227 (92.7%)	45 (83.3%)
CABG^c^:		317									
Yes	13 (4.10%)		6 (15.4%)	3 (2.19%)	4 (2.84%)	2 (5.71%)	10 (4.55%)	1 (1.61%)	1 (5.56%)	9 (3.67%)	3 (5.56%)
No	304 (95.9%)		33 (84.6%)	134 (97.8%)	137 (97.2%)	33 (94.3%)	210 (95.5%)	61 (98.4%)	17 (94.4%)	236 (96.3%)	51 (94.4%)
PCI^d^:		317									
Yes	34 (10.7%)		10 (25.6%)	5 (3.65%)	19 (13.5%)	4 (11.4%)	22 (10.0%)	8 (12.9%)	2 (11.1%)	19 (7.76%)	13 (24.1%)
No	283 (89.3%)		29 (74.4%)	132 (96.4%)	122 (86.5%)	31 (88.6%)	198 (90.0%)	54 (87.1%)	16 (88.9%)	226 (92.2%)	41 (75.9%)
SSBCT^e^:		317									
Yes	4 (1.26%)		1 (2.56%)	2 (1.46%)	1 (0.71%)	1 (2.86%)	2 (0.91%)	1 (1.61%)	1 (5.56%)	3 (1.22%)	0 (0.00%)
No	313 (98.7%)		38 (97.4%)	135 (98.5%)	140 (99.3%)	34 (97.1%)	218 (99.1%)	61 (98.4%)	17 (94.4%)	242 (98.8%)	54 (100%)
MACE^f^ six weeks:		317									
No	273 (86.1%)		25 (64.1%)	134 (97.82%)	114 (80.9%)	23 (65.7%)	195 (88.6%)	52 (83.9%)	12 (66.7%)	219 (89.4%)	39 (72.2%)
Yes	44 (13.8%)		14 (35.9%)	3 (2.18%)	27 (19.1%)	12 (34.3%)	25 (11.4%)	10 (16.1%)	6 (33.3%)	26 (10.6%)	15 (27.8%)

a: death due to any cause; b: acute myocardial infarction; c: coronary artery bypass grafting; d: percutaneous coronary intervention; e: significant coronary stenosis but conservative treatment; f: major adverse cardiovascular events.

The mean HEART score was4 in the PCEC (95% CI: 2.00–5.00) and the ED (95% CI: 3.00–6.00). The GRACE scale scored lower in the PCEC than in the ED 77 (95% CI: 59.5–101) vs 99 (95% CI: 77.0–126). The TIMI score obtained a 1 (95% CI: 0.00–2.00) in the PCEC and a 2 (95% CI: 2 1.00–3.00) in the ED.

The most frequent MACE were those that required PCI (10.7%;). The most frequent type of MACE in high-risk patients differed according to the risk stratification score: MACE requiring PCI, according to the HEART score (25.6% and DDTAC, according to the GRACE (17.1%) and TIMI (16.7%) scores (Table 2).

The balanced accuracy in the risk stratification of MACE in the low- and moderate–high risk patients was 0.67 (95% CI: 0.58–0.69) between the HEART and GRACE scores, 0.68 (95% CI: 0.59–0.69) between the HEART and TIMI scores, and 0.67 (95% CI: 0.67–0.77) between the TIMI and GRACE scores.

The ability of the HEART scale to diagnose MACE was very high, with an SE of 100% (95% CI: 100–100) and an NPV of 100% (95% CI:100–100) in low-risk patients, and an SP of 90.74% (95% CI:87.28–94.2) and a PPV of 88.13% (95% CI:84.33–91.93) in high-risk patients (Table 3).

**Table 3:** Performance characteristics of the TIMI, GRACE, and HEART risk scales.

	Accuracy(95%CI)	Sensitivity (95%CI)	Specificity (95%CI)	PPV (95%CI)	NPV (95%CI)	False Negative Rate	False positive rate
**TIMI score**							
TOTAL > = 1	41.96 (36.46–47.6)	91.49 (79.62–97.63)	33.33 (27.74–39.3)	19.28 (14.32–25.08)	95.74 (89.46–98.83)	8.51 (2.37–20.38)	66.67 (60.7–72.26)
TOTAL > = 2	61.83 (56.23–67.2)	78.72 (64.34–89.3)	58.89 (52.76–64.82)	25 (18.25–32.78)	94.08 (89.39–97.13)	21.28 (10.7–35.66)	41.11 (35.18–47.24)
TOTAL > = 3	75.71 (70.6–80.33)	44.68 (30.17–59.88)	81.11 (75.92–85.6)	29.17 (19.05–41.07)	89.39 (84.84–92.95)	55.32 (40.12–69.83)	18.89 (14.4–24.08)
TOTAL > = 5	83.28 (78.71–87.22)	12.77 (4.83–25.74)	95.56 (92.37–97.68)	33.33 (13.34–59.01)	86.29 (81.86–89.98)	87.23 (74.26–95.17)	4.44 (2.32–7.63)
**GRACE score**							
TOTAL > = 50	20.82 (16.48–25.71)	97.87 (88.71–99.95)	7.41 (4.58–11.21)	15.54 (11.61–20.18)	95.24 (76.18–99.88)	2.13 (0.05–11.29)	92.59 (88.79–95.42)
TOTAL > = 75	43.53 (38–49.19)	78.72 (64.34–89.3)	37.41 (31.62–43.48)	17.96 (12.97–23.9)	90.99 (84.06–95.59)	21.28 (10.7–35.66)	62.59 (56.52–68.38)
TOTAL > = 100	64.04(58.49–69.33)	57.45 (42.18–71.74)	65.19 (59.17–70.86)	22.31 (15.25–30.78)	89.8 (84.68–93.65)	42.55 (28.26–57.82)	34.81 (29.14–40.83)
TOTAL > = 109	68.45(63.03–73.53)	46.81 (32.11–61.92)	72.22 (66.47–77.48)	22.68 (14.79–32.3)	88.64 (83.68–92.51)	53.19 (38.08–67.89)	27.78 (22.52–33.53)
TOTAL > = 141	81.7 (77–85.8)	25.53 (13.94–40.35)	91.48 (87.49–94.52)	34.29 (19.13–52.21)	87.59 (83.16–91.2)	74.47 (59.65–86.06)	8.52 (5.48–12.51)
**HEART score**							
TOTAL > = 1	18.3 (14.2–23)	100 (92.45–100)	4.07 (2.05–7.17)	15.36 (11.51–19.9)	100 (71.51–100)	0 (0–7.55)	95.93 (92.83–97.95)
TOTAL > = 2	26.81 (22.02–32.05)	100 (92.45–100)	14.07 (10.16–18.8)	16.85 (12.65–21.76)	100 (90.75–100)	0 (0–7.55)	85.93 (81.2–89.84)
TOTAL > = 3	37.85 (32.49–43.45)	97.87 (88.71–99.95)	27.41 (22.18–33.14)	19.01 (14.27–24.53)	98.67 (92.79–99.97)	2.13 (0.05–11.29)	72.59 (66.86–77.82)
TOTAL > = 4	54.26 (48.6–59.84)	87.23 (74.26–95.17)	48.52 (42.42–54.65)	22.78 (16.87–29.61)	95.62 (90.71–98.38)	12.77 (4.83–25.74)	51.48 (45.35–57.58)
TOTAL > = 7	81.7 (77–85.8)	29.79 (17.34–44.89)	90.74 (86.64–93.92)	35.9 (21.2–52.82)	88.13 (83.73–91.69)	70.21 (55.11–82.66)	9.26 (6.08–13.36)

CI: confidence interval; PPV: positive predictive value; NPV**:** negative predictive value; TIMI score: low risk determined by score =  < 2, moderate risk determined by score =  < 4, and high risk determined by score >  = 5; GRACE score: low risk determined by score <  = 108, moderate risk determined by score >  = 109, and high risk determined by score >  = 141; HEART score: low risk determined by score =  < 3, moderate risk determined by score >  = 4, and high risk determined by score >  = 7.

Regarding the likelihood of MACE occurring, the HEART score performed significantly better than the TIMI and GRACE scores: the risk of MACE in moderate-risk patients was 5 times higher than in low-risk patients (OR 5.32[95% CI: 2.07–15.79]); Table 4).

**Table 4:** Comparison of the Odds Ratio—MACE according to the risk stratification of the HEART, GRACE, and TIMI scales.

	Odds ratio (95% CI)	Hosmer–Lemeshow statistic	Hosmer–Lemeshow p-value	Adjusted odds ratio (95% CI)
HEART-High vs Low	12.227 (4.465—37.432)	0	1	12.219 (3.894—42.929)
HEART-Moderate vs Low	5.171 (2.196—14.254)			5.322 (2.073—15.793)
GRACE- High vs Low	4.07 (1.771—9.105)	0	1	3.121 (1.02—9.645)
GRACE-Moderate vs Low	1.5 (0.651—3.24)			1.349 (0.481—3.669)
TIMI- High vs Low	4.212 (1.37—11.851)	0	1	3.38 (1.024—10.423)
TIMI-Moderate vs Low	3.24 (1.551—6.616)			2.684 (1.19—5.971)

CI: confidence interval.

**Figure 3:**
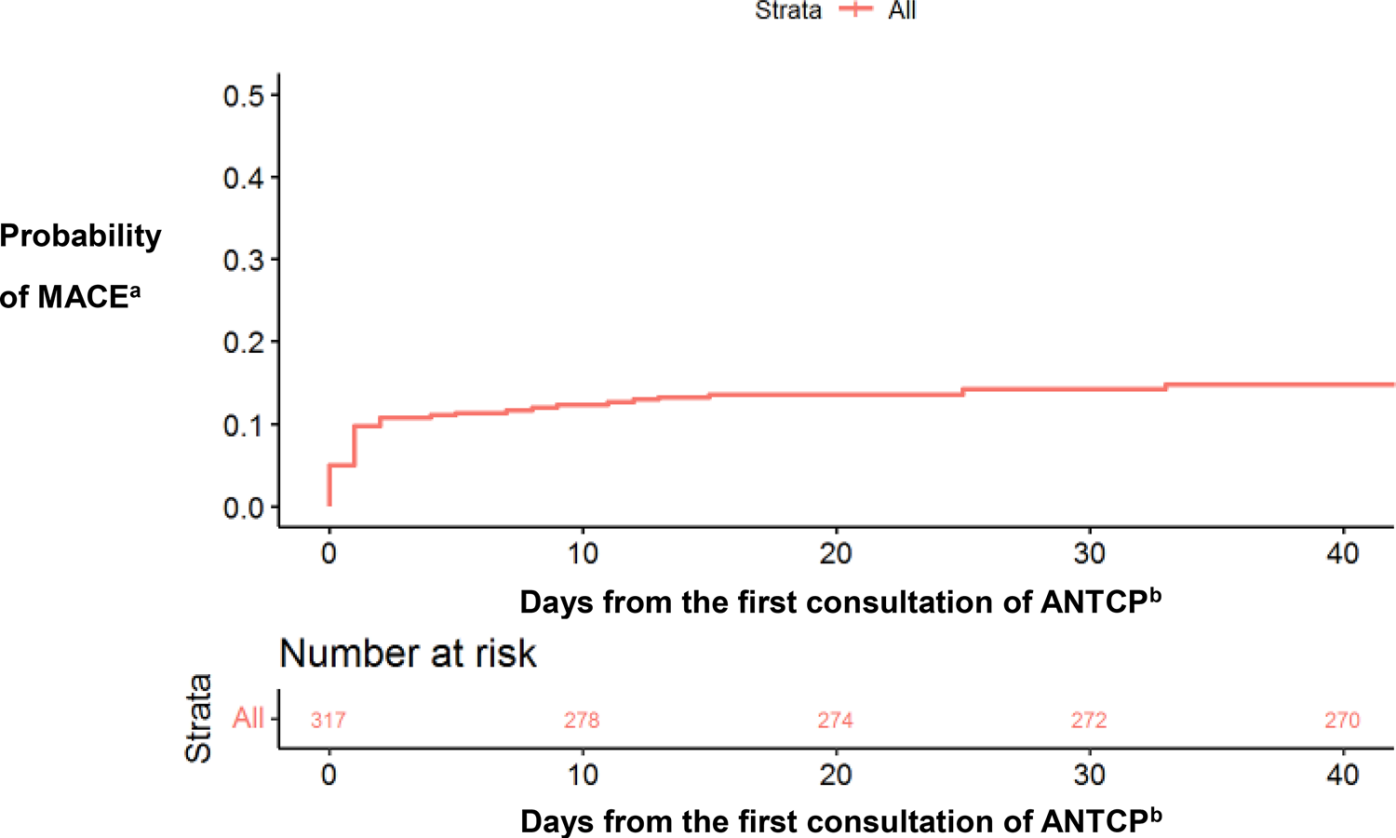
Survival analysis: global Kaplan Meier curve.a: major acute cardiac event; b:acute non-traumatic chest pain.

The mean time to MACE was one day (95% CI:0.00–8). The mean time to DDTAC was nine days (95% CI: 7.50–24), to AMI was one day (95% CI: 0.00–1), to PCI was one day (0.25–9.50), and to CABG was one day (95% CI: 0.25–8.75) (Fig. 3).

The kappa index in the categorical classification of patients as low- and moderate–high-risk was 0.32 (95% CI: 0.58–0.69) according to the HEART and GRACE scores, according to the HEART and TIMI scores, and 0.31 (95% CI: 0.67–0.77) according to the GRACE and TIMI scores.

## Discussion

In this study, we compared the effectiveness of the HEART, GRACE, and TIMI scales for the diagnosis and prognosis of MACE at six weeks in patients with ANTCP and suspected NSTE-ACS. The HEART scale was superior to TIMI and GRACE, with results similar to previous studies^10,11,21^. Specifically, the HEART scale is more effective in stratifying low-risk patients, with an excellent level of safety. In this group, 2.18% of patients (95% CI 1.2–3.2) had MACE at six weeks, similar to the 2.5% observed by Six et al.^7^, and our own previous observations from a retrospective study^15^.

The HEART score is currently validated for use in some medical emergency services, and it is considered superior to TIMI and GRACE (GRACE 2.0) for determining MACE risk at six weeks, with highSE and a NPV^9,11,26^. Poldervaart et al.^10^ observed that the HEART score identified low-risk patients better than the TIMI and GRACE scores, with only a 0.8% incidence of MACE in this group of patients. Stopyra et al.^14^ integrated the HEART score with the clinical history of patients and observedthat 97.5% of low-risk patients did not die of AMI within one year, with a 7% reduction in the yearly hospitalization rate.

The superior diagnostic and predictive ability for MACE at six weeks of the HEART score compared to the TIMI and GRACE scores was expected. Indeed, HEART was designed to predict MACE at six weeks in patients with ANTCP^7,9^, while TIMI and GRACE were created to determine the prognosis of patients with unstable angina and NSTE-ACS and are not effective for patients with a medium–low probability of MACE^19^. Therefore, the efficacyof the TIMI and GRACE scores in predicting MACE is questioned and subjected to constant validity studies^14,19,21,26^.

The higher the SE and NPV, the better the clinical scoring system for risk stratification^22^. In our study, the HEART score stratified low-risk patients for MACE at six weeks better than the TIMI and GRACE scores, with the best SE and NPV. Similar results were obtained in previous validation studies, in which the SE ranged from 99% (95% CI 97%–100%) to 99.5% (95% CI 97.1%–99.9%) and the NVP between 99% (98%–100%) and 99.6% (95% CI 97.3%–99.9%) ^9–11,14,19^.

We also observed a higher AUC and a better PPV in high-risk patients (35.9% [95% CI: 20.84–50.95]) for the HEART score compared to the TIMI and GRACE scores, reinforcing the superior diagnostic performance of this clinical tool. Similar results were obtained by others for the AUC (0.83 [95% CI 0.81–0.85]) for HEART regarding MACE at 30 days^9^ and PPV (36%[30%–41%]–46% [40%–52%])^8,9,19^.

Kline JA et al.^21^ evaluated the risk score for MACE at 30 days for chest pain, suggesting arange of0.5%–3.0% for a valid FNR, with an average estimationof 2.0% for low-risk patients. Wamala et al.^19^ compared nine coronary risk scores and alsoobtained an FNR of 2% for the HEART score in low risk patients, while Stark et al.^22^ found an FNR of 0.09% to 3.2% for low-riskMACE patients according to the HEART scale. We obtained similar results, with an FNR of 0 to 2.13% for low-risk MACE patients according to the HEART scale.Additionally, the OR and the Kapplan Meir we obtained highlight the better risk stratification by the HEART score and are similar to that of Six et al.^7^.

Scales that do not include chest pain would be expected to have better reproducibility and reliability and less interobserver bias y^12^. On the contrary, we observed that chest pain is a relevant component and does not reduce the effectiveness of the HEART score, with each of its variables being statistically significant (*p* < 0.001).

The kappa index between the HEART, GRACE, and TIMI scores was acceptable in the categorical classification of patients as low- and moderate–high-risk, which reinforces the high degree of reliability of the HEART scale.

The effective risk stratification of ANTCP patients in EDs and PCECs is always a challenge. Recent attempts to further improve the accuracy of predictive models include more specific cardiac markers and the combination of risk scores and other complementary tests that are not present in some hospital EDs and most PCECs. In this sense, the HEART score is easier to calculate compared to other risk scales because its elements are more affordable. From the GRACE score, the Killip class component requires the physician’s judgment for the diagnosis of heart failure, generating inter-rater variation. Very few patients undergo additional tests for heart failure, such as ECG and natriuretic peptide level, because they are not available in PCECs and some hospital EDs. In our study, only 1.2% of the patients assisted in the HUAV ED had their natriuretic peptide level measured, and 31.9% underwent an ECG.In the CUAP, none of the patients underwent these tests because the resources were not available.

### Limitations

Although we obtained a representative sample that allows our results to be extrapolated, we included only one ED and one PCEC, with their own care and population characteristics. Therefore, our findings should be interpreted with care. A study including more EDs and PCECs is needed to support these data.

## Conclusions

The HEART score is better than the TIMI and GRACE scores for the diagnosis and prognosis of MACE at six weeks for low-risk patients in Spain attending PCECs and EDs with ANTCP and suspected NSTE-ACS.This eliminates the need for more complementary tests without compromising patient health.

## Data Availability

The datasets used and/or analyzed during the current study are available from the corresponding author on reasonable request.

## Abbreviations

ACS: Acute coronary syndrome
AMI: Acute myocardial infarction
ANTCP: Acute non-traumatic chest pain
AUC: Area under the curve
CABG: Coronary artery bypass grafting
CC: Cardiac cause
CK: Creatine kinaseNI
CK-MB: Creatine kinase myocardial band
cTnT: Cardiac troponin T
CUAP: Primary care emergency center
DD: D-dimers
ECAP: Electronic medical records of the patients from the Catalan Health Institute’s database
ICS: Lleida
ECG: Electrocardiogram
ED: Emergency department
ESC: European Society of Cardiology
FNR: False negative rate
FPR: False positive rate
GRACE score: Global registry of acute coronary events
HEART: History, ECG, age, risk factors and troponin
HUAV: Hospital Universitari Arnau de Vilanova
ICD-10: International Statistical Classification of Diseases and Related Health Problems 10th Revision.
ICS: InstitutCatalà de la Salut
MACE: Major adverse cardiac event
MR: Magnetic resonance
NICE: National Institute for Health and Care Excellence
NPV: Negative predictive value
NSTE-ACS: Acute coronary syndrome without ST segment elevation
NSTEMI: Non ST elevation myocardial infarction
PCEC: Primary care emergency center
PCI: Percutaneous coronary intervention
PCR: C-reactive protein
PPV: Positive predictive value
pro-BNP: B-type natriuretic peptide
RED Cap: Research Electronic Data Capture web platform
ROC: Receiver operating characteristic
SAP: System—administrative management of patients
SCSBCT: Significant coronary stenosis but conservative treatment
SE: Sensitivity
SP: Specificity
TAC: Computed axial tomography
TIMI score: Thrombolysis in myocardial infarction
TnI: Troponin I
